# Supplementary material for: Detection and prevalence of a novel Bandavirus related to Guertu virus in Amblyomma gemma ticks and human populations in Isiolo County, Kenya
Source: PLoS One. 2024 Sep 20;19(9):e0310862. doi: 10.1371/journal.pone.0310862 (PMC11414941; doi:10.1371/journal.pone.0310862)
Supplement: S1 Table — (DOCX) [file pone.0310862.s001.docx]

| **Primer Name** | **Sequence** | **Size** | **Target** |
| --- | --- | --- | --- |
| BCS82C | ATG ACT GAG TTG GAG TTT CAT GAT GTC GC | 251 bp | California Bunyamwera |
| BCS332V | TGT TCC TGT TGC CAG GAA AAT |  |  |
| VIR2052F | TGG CGC TAT GAT GAA ATC TGG AAT GTT | 150bp | Alphavirus |
| VIR2052R | TAC GAT GTT GTC GTC GCC GAT GAA- |  |  |
| FU 1 | TAC AAC ATG ATG GGA AAG AGA GAG AA | ~260bp | Flavivirus |
| CFD2 | GTG TCC CAG CCG GCG GTG TCA TCA GC |  |  |
| DHO-NR2F | TGGTACCCTTTTCTTGCTTCACTCC | 652bp | Dhori |
| DHO-NF2R | TGGACACCTTCACAAACTC |  |  |
| CCHF F2 | GACAAATTCCCTGCACCA | 536bp | CCHFV |
| CCHF R3 | GGG TAC TGA TCA TAG CCG TAG |  |  |
| THO-PB-334F | GCACGCCTGAAGTAGGTATGT | 327bp | Thogoto |
| THO-PB-8R | TTGCAGATGTAATGGAAATGG |  |  |
| DgM058F | CTC TAA GCC CGA GGA CCC TG | 563bp | Dugbe |
| Dg M621W | GGTCAACAAATCATAAAGATATTGG |  |  |
