# Supplementary material for: Detection and prevalence of a novel Bandavirus related to Guertu virus in Amblyomma gemma ticks and human populations in Isiolo County, Kenya
Source: PLoS One. 2024 Sep 20;19(9):e0310862. doi: 10.1371/journal.pone.0310862 (PMC11414941; doi:10.1371/journal.pone.0310862)
Supplement: Attachment — Submitted filename: PONE-D-24-20220_SBM.pdf [file pone.0310862.s002.pdf]

PLOS ONE

Detection and prevalence of a novel Bandavirus related to Guertu virus in Amblyomma gemma ticks and human populations in Isiolo County, Kenya

--Manuscript Draft--

**Manuscript Number:**	PONE-D-24-20220
**Article Type:**	Research Article
**Full Title:**	Detection and prevalence of a novel Bandavirus related to Guertu virus in Amblyomma gemma ticks and human populations in Isiolo County, Kenya
**Short Title:**	Isolation of a novel Bandavirus closely related to Guertu virus
**Corresponding Author:**	Hellen Koka, MSc<br>Kenya Medical Research Institute<br>Nairobi, Nairobi KENYA
**Keywords:**	Ticks; novel virus; tick-borne viruses; Guertu virus; Phenuiviridae
**Abstract:**	Emerging tick-borne viruses of medical and veterinary importance are increasingly being reported globally. This resurgence emphasizes the need for sustained surveillance to provide insights into tick-borne viral diversity and associated potential public health risks. We report on a virus tentatively designated Kinna virus (KIV) in the family Phenuiviridae and genus Bandavirus. The virus was isolated from a pool of Amblyomma gemma ticks from Kinna in Isiolo County, Kenya. High throughput sequencing of the virus isolate revealed close relatedness to the Guertu virus. The virus genome is consistent with the described genomes of other members of the genus Bandavirus, with nucleotides lengths of 6403, 3332 and 1752 in the Large (L), Medium (M) and S (Small) segments respectively. Phylogenetic analysis showed that the virus clustered with Guertu virus although it formed a distinct and well supported branch. The RdRp amino acid sequence had a 93.3 % identity to that of Guertu virus, an indication that the virus is possibly novel. Neutralizing antibodies were detected in 125 (38.6%, 95% CI 33.3–44.1%) of the human sera from the communities in this region. In vivo experiments showed that the virus was lethal to mice with death occurring 6-9 days post infection. The virus infected mammalian cells (Vero cells) but had reduced infectivity in the mosquito cell line (C636) tested.<br>Conclusion<br>Isolation of this novel virus with the potential to cause disease in human and animal populations necessitates the need to evaluate its public health significance and contribution to disease burden in the affected regions. This also points to the need for continuous monitoring of vector and human populations in high-risk ecosystems to update pathogen diversity.
**Order of Authors:**	Hellen Koka, Msc
	Solomon Langat, MSc
	Samuel Oyola, PhD
	Gilbert Rotich, BSc
	Faith Cherop, BSc
	James Mutisya, MSc
	Victor Ofula, MSc
	Konongoi Limbaso, DVM, MSc
	Juliette R Ongus, PhD
	Joel Lutomiah, PhD
	Rosemary Sang, PhD
**Additional Information:**	
**Question**	**Response**

Powered by Editorial Manager® and ProduXion Manager® from Aries Systems Corporation

**Financial Disclosure**<br><br>Enter a financial disclosure statement that describes the sources of funding for the work included in this submission. Review the submission guidelines for detailed requirements. View published research articles from *PLOS ONE* for specific examples.<br><br>This statement is required for submission and **will appear in the published article** if the submission is accepted. Please make sure it is accurate.<br><br>**Funded studies**<br>Enter a statement with the following details:<br>• Initials of the authors who received each award<br>• Grant numbers awarded to each author<br>• The full name of each funder<br>• URL of each funder website<br>• Did the sponsors or funders play any role in the study design, data collection and analysis, decision to publish, or preparation of the manuscript?<br><br>Did you receive funding for this work?	Yes
Please add funding details.<br>as follow-up to "**Financial Disclosure**<br><br>Enter a financial disclosure statement that describes the sources of funding for the work included in this submission. Review the submission guidelines for detailed requirements. View published research articles from *PLOS ONE* for specific examples.<br><br>This statement is required for submission and **will appear in the published article** if the submission is accepted. Please make sure it is accurate.	This study was partially funded by a grant from Mawazo Institute (https://mawazoinstitute.org), grant number 2022-1-17 and support from the government of Kenya through the Kenya Medical Research Institute. The views expressed herein do not necessarily reflect the official opinion of the donors. The funders had no role in the design of the study; in the collection, analyses, or interpretation of data; in the writing of the manuscript; or in the decision to publish the results.

Powered by Editorial Manager® and ProduXion Manager® from Aries Systems Corporation

**Funded studies**<br>Enter a statement with the following details:<br>• Initials of the authors who received each award<br>• Grant numbers awarded to each author<br>• The full name of each funder<br>• URL of each funder website<br>• Did the sponsors or funders play any role in the study design, data collection and analysis, decision to publish, or preparation of the manuscript?<br><br>Did you receive funding for this work?"	
Please select the country of your main research funder (please select carefully as in some cases this is used in fee calculation).<br>as follow-up to "**Financial Disclosure**<br><br>Enter a financial disclosure statement that describes the sources of funding for the work included in this submission. Review the submission guidelines for detailed requirements. View published research articles from *PLOS ONE* for specific examples.<br><br>This statement is required for submission and **will appear in the published article** if the submission is accepted. Please make sure it is accurate.<br><br>**Funded studies**<br>Enter a statement with the following details:<br>• Initials of the authors who received each award<br>• Grant numbers awarded to each author<br>• The full name of each funder<br>• URL of each funder website<br>• Did the sponsors or funders play any role in the study design, data collection and analysis, decision to publish, or preparation of the manuscript?<br><br>Did you receive funding for this work?"	KENYA - KE
**Competing Interests**	The authors declare that they have no competing interests

Powered by Editorial Manager® and ProduXion Manager® from Aries Systems Corporation

Use the instructions below to enter a competing interest statement for this submission. On behalf of all authors, disclose any competing interests that could be perceived to bias this work—acknowledging all financial support and any other relevant financial or non-financial competing interests.<br><br>This statement is required for submission and **will appear in the published article** if the submission is accepted. Please make sure it is accurate and that any funding sources listed in your Funding Information later in the submission form are also declared in your Financial Disclosure statement.<br><br>View published research articles from *PLOS ONE* for specific examples.<br><br>**NO authors have competing interests**<br><br>Enter: *The authors have declared that no competing interests exist.*<br><br>**Authors with competing interests**<br><br>Enter competing interest details beginning with this statement:<br><br>*I have read the journal's policy and the authors of this manuscript have the following competing interests: [insert competing interests here]*<br><br>* typeset	
**Ethics Statement**<br><br>Enter an ethics statement for this submission. This statement is required if the study involved:<br><br>• Human participants<br>• Human specimens or tissue<br>• Vertebrate animals or cephalopods<br>• Vertebrate embryos or tissues	Scientific and ethical approval for the study protocol and animal use was obtained from the Kenya Medical Research Institute, Scientific and Ethics Review Unit and the institute's Animal Care and Use Committee(KEMRI/SERU/CVR/007/4514).

• Field research<br><br>Write "N/A" if the submission does not require an ethics statement.<br><br>General guidance is provided below. Consult the submission guidelines for detailed instructions. **Make sure that all information entered here is included in the Methods section of the manuscript.**	

**Format for specific study types**<br><br>**Human Subject Research (involving human participants and/or tissue)**<br>• Give the name of the institutional review board or ethics committee that approved the study<br>• Include the approval number and/or a statement indicating approval of this research<br>• Indicate the form of consent obtained (written/oral) or the reason that consent was not obtained (e.g. the data were analyzed anonymously)<br><br>**Animal Research (involving vertebrate animals, embryos or tissues)**<br>• Provide the name of the Institutional Animal Care and Use Committee (IACUC) or other relevant ethics board that reviewed the study protocol, and indicate whether they approved this research or granted a formal waiver of ethical approval<br>• Include an approval number if one was obtained<br>• If the study involved *non-human primates*, add *additional details* about animal welfare and steps taken to ameliorate suffering<br>• If anesthesia, euthanasia, or any kind of animal sacrifice is part of the study, include briefly which substances and/or methods were applied<br><br>**Field Research**<br><br>Include the following details if this study involves the collection of plant, animal, or other materials from a natural setting:<br>• Field permit number<br>• Name of the institution or relevant body that granted permission	
**Data Availability**<br><br>Authors are required to make all data underlying the findings described fully available, without restriction, and from the time of publication. PLOS allows rare exceptions to address legal and ethical concerns. See the PLOS Data Policy and FAQ for detailed information.	Yes - all data are fully available without restriction

A Data Availability Statement describing where the data can be found is required at submission. Your answers to this question constitute the Data Availability Statement and **will be published in the article**, if accepted.<br><br>**Important:** Stating 'data available on request from the author' is not sufficient. If your data are only available upon request, select 'No' for the first question and explain your exceptional situation in the text box.<br><br>Do the authors confirm that all data underlying the findings described in their manuscript are fully available without restriction?	
**Describe where the data may be found in full sentences. If you are copying our sample text, replace any instances of XXX with the appropriate details.**<br><br>• If the data are **held or will be held in a public repository**, include URLs, accession numbers or DOIs. If this information will only be available after acceptance, indicate this by ticking the box below. For example: *All XXX files are available from the XXX database (accession number(s) XXX, XXX.).*<br>• If the data are all contained **within the manuscript and/or Supporting Information files**, enter the following: *All relevant data are within the manuscript and its Supporting Information files.*<br>• If neither of these applies but you are able to provide **details of access elsewhere**, with or without limitations, please do so. For example:<br><br>*Data cannot be shared publicly because of [XXX]. Data are available from the XXX Institutional Data Access / Ethics Committee (contact via XXX) for researchers who meet the criteria for access to confidential data.*<br><br>*The data underlying the results presented in the study are available from (include the name of the third party*	All data generated or analyzed in this study are included in this published article and its supplementary files. Accession numbers for the L, M and S segments of the virus are PP580190-PP580192 respectively.

and contact information or URL).<br>• This text is appropriate if the data are owned by a third party and authors do not have permission to share the data.<br><br>* typeset	
Additional data availability information:	Tick here if the URLs/accession numbers/DOIs will be available only after acceptance of the manuscript for publication so that we can ensure their inclusion before publication.

Powered by Editorial Manager® and ProduXion Manager® from Aries Systems Corporation

Manuscript

Click here to access/download;Manuscript;Tick borne viruses Guertu-like edits May 24 2024 Plos one.docx

Full title: Detection and prevalence of a novel *Bandavirus* related to Guertu virus in *Amblyomma gemma* ticks and human populations in Isiolo County, Kenya

Short title: Isolation of a novel *Bandavirus* closely related to Guertu virus

Authors

Hellen Koka[1, 2*], Solomon Langat[1], Samuel Oyola[4], Faith Cherop[3], Gilbert Rotich[3], James Mutisya[1], Victor Ofula[1], Konongoi Limbaso[1], Juliette R. Ongus[2], Joel Lutomiah[1], Rosemary Sang[3]

*Corresponding author: hellenkoka@gmail.com

Affiliations

[1] Kenya Medical Research Institute, Centre for Virus Research, P. O. Box 54840-00200 Nairobi, Kenya;

[2] Jomo Kenyatta University of Agriculture and Technology, P.O. Box 62000-00200, Nairobi, Kenya;

[3] International Centre of Insect Physiology and Ecology, P.O. Box 30772-00100, Nairobi, Kenya

[4] International Livestock Research Institute, P. O. Box 30709-00100, Nairobi, Kenya

Abstract

Introduction

Emerging tick-borne viruses of medical and veterinary importance are increasingly being reported globally. This resurgence emphasizes the need for sustained surveillance to provide insights into tick-borne viral diversity and associated potential public health risks. We report on a virus tentatively designated Kinna virus (KIV) in the family *Phenuiviridae* and genus *Bandavirus*. The virus was isolated from a pool of *Amblyomma gemma* ticks from Kinna in Isiolo County, Kenya. High throughput sequencing of the virus isolate revealed close relatedness to the Guertu virus. The virus genome is consistent with the described genomes of other members of the genus *Bandavirus,* with nucleotides lengths of 6403, 3332 and 1752 in the Large (L), Medium (M) and S (Small) segments respectively. Phylogenetic analysis showed that the virus clustered with Guertu virus although it formed a distinct and well supported branch. The RdRp amino acid sequence had a 93.3 % identity to that of Guertu virus, an indication that the virus is possibly novel. Neutralizing antibodies were detected in 125 (38.6%, 95% CI 33.3–44.1%) of the human sera from the communities in this region. In vivo experiments showed that the virus was lethal to mice with death occurring 6-9 days post infection. The virus infected mammalian cells (Vero cells) but had reduced infectivity in the mosquito cell line (C636) tested.

Conclusion

Isolation of this novel virus with the potential to cause disease in human and animal populations necessitates the need to evaluate its public health significance and contribution to disease burden in the affected regions. This also points to the need for continuous monitoring of vector and human populations in high-risk ecosystems to update pathogen diversity.

Key words

Ticks, novel virus, tick-borne viruses, Guertu virus, *Phenuiviridae*

Introduction

The incidence of tick-borne diseases is increasing globally and has been attributed to multiple factors including climate change that have led to the expansion of tick vectors into new geographical habitats (1). Tick-borne viruses belong to nine families: *Asfaviridae, Flaviviridae, Reoviridae, Orthomoxyviridae, Rhabdoviridae, Nyamiviridae, Phenuiviridae, Nairoviridae* and *Peribunyaviridae* (2) with over 160 viruses that affect humans and animals (3). Some of the newly identified tick-borne viruses include *Dabie bandavirus* also known as Severe Fever Thrombocytopenia Syndrome Virus (SFTSV) in China, Heart land virus and the Deer tick Virus in North America, Kyasanur Forest Disease in India and Alkhurma Hemorrhagic Fever in Saudi Arabia (4). The unprecedented number of novel tick-borne viruses being reported, can also be attributed to technological advances in molecular biology (5). Most of the novel viruses have been placed in established genera although some of the viruses are yet to be assigned to a family or genera (6). Currently, the genus *Bandavirus* as indicated in the International Committee on Taxonomy of Viruses (ICTV) 2021 release, consists of eight tick-borne bunyaviruses in the order *Bunyavirales* and family *Phenuiviridae.* These include *Dabie bandavirus, Bhanja bandavirus, Guertu bandavirus, Heartland bandavirus, Hunter Island bandavirus, Kismaayo bandavirus, Lone Star bandavirus* and *Razdan bandavirus* (7). The representative species are *Dabie bandavirus* (SFTSV), Bhanja virus (BHAV), Guertu virus (GTV), Heartland virus (HRTV), Hunter Island virus (HUIV), Kismaayo Virus (KISV), Lone star virus (LSV) and Razdan virus (8).

Dabie bandavirus (SFTSV), an emerging tick-borne hemorrhagic fever virus, was first isolated in patients from rural China in 2009 (9) and has since been reported in several Asian countries including Japan, South Korea and Vietnam (10-12). This virus is associated with high fatality

rates of 12-50% with clinical symptoms that include fever, myalgia, gastrointestinal symptoms and laboratory abnormalities such as regional lymphadenopathy, thrombocytopenia, leucopenia and elevated serum hepatic enzymes (13). Severe cases progress to hemorrhagic manifestations, neurologic symptoms followed by multiple organ failure and death, with the most vulnerable being the elderly and immunocompromised individuals (14). Although nosocomial infections were reported in China through contact with infected blood or mucus, the virus is mainly transmitted by ticks (15, 16). In China, the virus was detected in *Rhipicephalus microplus* and *Haemaphysalis longicornis* ticks, while transtadial as well as transovarial transmission has been documented (17). Various wild and domestic animals such as deer, rodents, boars, cattle, goats, pigs, dogs and birds are also implicated in the transmission cycle of this virus with goats and sheep showing high sero-prevalence rates (18, 19). In the United States, *Amblyomma americanum* have been implicated in the transmission of this virus (20). Another novel virus, Heartland Virus, that is genetically related to SFTSV was also reported in the United States in 2009, in patients hospitalized with fever, fatigue, anorexia and diarrhea with laboratory abnormalities similar to those of SFTSV (21). Guertu virus, also a novel highly pathogenic bunyavirus, was first isolated in 2014 from *Demarcentor nuttalli* ticks in the Guertu mountain region of Wusu Xinjiang in China (22, 23). It is genetically related to the SFTSV and Heartland virus, with up to 90% sequence identity to SFTSV. Guertu virus infects animal and human cell lines and is pathogenic to mice (23). Antibodies with neutralizing activity against Guertu virus were detected in human serum samples in the Wusu region indicating the viruses' potential to infect humans (23). Guertu virus, like all *Bandaviruses,* has a tripartite RNA genome consisting of the large (L), Medium (M) and Small (S) segments. The L segment contains one large open reading frame (ORF) which encodes for the RNA-dependent RNA polymerase (RdRp) gene. The

M segment also contains a single ORF that encodes for the glycoprotein precursor (comprising Gn and Gc genes), while the S segment contains two ORFs that encodes for the nucleocapsid (N) and nonstructural proteins (NSs) which exhibits an ambisense gene organization (24). Several tick-borne viruses including Guertu, were reported in *Hyalomma* ticks from Saudi Arabia (25). Neutralizing antibodies to Guertu virus were also reported in human samples from Pakistan (26). Recent reports of Jingmen tick virus in Kenya, underscore the need for continued surveillance of tick-borne viruses and an assessment of the risk they pose to the public health in pastoralist communities (27, 28). Pastoralist communities live in dry land systems and the livestock interface with wildlife in search of pasture (29) posing a risk for diseases transmission (30). This study describes a virus that was isolated from *A. gemma* ticks collected from domestic animals in pastoralist communities in Isiolo County, Kenya. Full genome sequence analysis determined the virus to be closely related to Guertu virus and herein we provide data on its phylogeny and sero-prevalence in humans in this dry land ecosystem.

Materials and methods

Study site

Isiolo County is an arid and semi-arid area with annual rainfall ranges of 150mm-600mm and temperatures range of 24^0C to 30^0C. The land is covered by bushlands, scrubs and shrubs that are utilized as pastoral grazing land and managed by conservancies in the community led by a board of local trustees. Wildlife and tourism are fundamental to this county as it has many wildlife reserves such as Shaba, Buffalo springs, Bisanadi. On the border of Isiolo and Meru county is Meru national park that is close to some of these villages in this study (31). Farming is limited to areas around Isiolo central and Kinna that are close to the Ewaso Nyiro River and other tributaries such as the Ngare Mara and Kinna among others.

Study populations

Ticks were sampled from four sites in Isiolo County, namely Kinna, Kulamawe, Garbartula and Merti from 2015-2017. Ticks were collected from different domestic animal hosts in September 2015, April 2016 and December 2017. All ticks from one animal per site were stored in 50 ml centrifuge tubes and preserved in liquid nitrogen shippers for transportation to the laboratory. The archived human samples used in this study were collected in 2015 during a community-based surveillance study targeting healthy individuals from five villages namely: - Kinna, Garfasa, Dadachabasa, Korbesa and Bulesa in Merti and Garbatula districts. The study sought to determine human exposure to arboviruses such as Rift Valley Fever Virus in nomadic populations along animal migratory routes at points of human-livestock and wildlife convergence (KEMRI SSC No.2346) (32). **The samples were used retrospectively in this study and were accessed on 11th November 2022. The data had been de-identified and thus authors could not identify individual participants during or after data collection**. The explanatory variables for this study were sex, age, occupation, contact with animals such as cows, goats, donkeys, camels and previous illness that was severe and/or haemorrhagic **(Fig 1)**.

what was the nature of the sample? I am assuming these are serum? please specify.

change to regular font

Fig 1. Map of Isiolo County showing the sites where tick and human samples were collected in this study. These maps were generated using ArcGIS version 10.2.2 for Desktop (Advanced License) courtesy of Samuel Owaka.

Laboratory procedures

Tick identification

Ticks were identified to species using available morphological keys (33, 34) and pooled (1-8 ticks per pool). Several (10-12) 2 mm Zicornia beads were added to each tick pool in a 1.5ml

centrifuge tube. The centrifuge tubes were frozen before tick homogenization using the Omni bead ruptor-24 at a speed of 3.7m/s for 1 minute. Homogenizing media prepared by supplementing Minimum Essential Medium (MEM) with 15% Fetal Bovine Serum (FBS), 2% L- glutamine, 2% antibiotic-antimycotic solution (100 units/ml penicillin, 100µg/ml streptomycin and amphotericin) was added to the tubes of crushed ticks, vortexed, followed by centrifugation at 10,000rpm for 5 minutes. The supernatants were transferred to cryovials for storage at -80^{0} C until samples were assayed.

Virus isolation by cell culture and primer design

Vero CCL-81 (ATTC) cell lines maintained in an incubator at 37°C with 5% CO_2 (Sanyo inCu safe, Japan) were used in virus isolation. The cell lines were seeded in 24-well plates and grown to 80-90% confluence in Minimum Essential Medium (MEM) supplemented with 10% Fetal Bovine Serum (FBS), 2% L- glutamine, 2% antibiotic-antimycotic solution (100 units/ml penicillin, 100µg/ml streptomycin and amphotericin) and 2ml of Non-Essential Amino Acid (NEAA). After inoculation with the tick supernatant, the cell lines were grown in MEM supplemented with 2% FBS, 2% L-glutamine, 2% antibiotic-antimycotic solution and 2ml NEAA. Cells were observed daily for viral Cytopathic Effect (CPE) for a maximum of 14 days. Tick supernatants were harvested upon observation of CPE causing 50-70% cell destruction. All the harvested tick pools were passaged to improve chances of virus recovery (35). The harvested virus isolates were subjected to a PCR using universal primers targeting flaviviruses, orthobunyaviruses and alphaviruses(36) as well as tick-borne viruses (37). Virus isolates that could not be identified using available primers were sequenced and new primers were designed from the sequences of the novel virus using the PrimerQuest tool (IDT technologies). PCR products were separated on a 2% agarose gel using Diamond nucleic acid dye (Promega).

Sequencing and bioinformatics analysis

The tick supernatant with viral CPE was harvested, centrifuged and then filtered using a 2.0mm filter to remove cell debris. RNA was extracted from the supernatant using the QIAamp Viral RNA mini kit (Qiagen, Germany), according to the manufacturer's protocol. RNA was quantified using the Qubit 2.0 fluorometer using the Qubit RNA HS assay kit (Invitrogen, USA). RNA libraries were prepared using the TruSeq mRNA Library Prep kit (Illumina, San Diego, CA, USA), following the manufacturer's recommended protocol which was modified to exclude the mRNA clean-up steps (38). The libraries were sequenced on an Illumina Miseq platform (Illumina, San Diego, CA, USA) using a 2x300 base paired-end reads. Raw sequence reads were inspected for quality using FastQC and subsequently filtered with Prinseq Lite v0.20.4. Cleaned paired reads were assembled *de novo* using Megahit v1.1.2 and the generated contigs were compared to NCBI nucleotide database using a cut-off E-value < 10^{-5}.

Phylogenetic analysis was performed using MEGA7 (39). Representative reference genomes were downloaded from GenBank through the NCBI Virus portal and combined with the sequence of the virus generated in this study. Alignment of the combined sequences was achieved with Muscle plugin embedded in MEGA7. Maximum Likelihood phylogenetic analysis was carried out with a bootstrap of 1000 replicates.

RNA extraction and RT-PCR

RNA was extracted from 200 tick pool samples from the different sites and the CPE-positive tick isolate was used as a control. Thereafter, the samples were screened for the novel virus using the new primers and superscript III One-step RT-PCR system with platinum taq High Fidelity DNA polymerase according to the manufacturer's instruction (Invitrogen). The following cycling conditions were used with an initial cDNA pre-denaturation of 50^0C for 20 minutes, followed by

94^0C 2 minutes and 40 cycles of 94^0C for 15 seconds, 62^0C for 30 seconds, 68^0C for 1 minutes and 68^0C for 5 minutes. The PCR products were viewed on a 2% gel.

In vitro virus growth kinetics

Vero E6, Vero Biken (JCRB0111), Vero CCL-81 and C6/36 cells seeded at $2.8x10^6$ cell/ml were infected with the virus ($5.3 x10^6$ PFU/ml) at a multiplicity of infection (MOI) of 1. After every 24 hours for 7 days, 200ul of cell culture supernatant was harvested from every infected cell line. Viral titres in these supernatants were quantified by plaque assay and a one-step growth curve generated.

In vivo studies in mice

This study was carried out following the recommendations in the Guide for the Care and Use of Laboratory Animals. The protocol was approved by the KEMRI Animal Care and Use Committee (KEMRIACUC/02.07.2023). Three female lactating mice each with a litter of 6-10 Swiss Albino mice (1-2 day-old) per cage from the KEMRI animal house were used in this study to determine virus pathogenicity. A 10-fold dilution of the virus was done and 0.01ml of the neat virus, 10^{-2} and 10^{-4} virus dilution were inoculated intracerebrally into the cranium of 1–2-day-old mice in a class II type A2 bio safety cabinet (40). All the litters of one female were inoculated with the same virus dose. A second replicate of mouse brain inoculations was done with the three virus dilutions but with different batches of litters. **A total of 22 suckling mice were used in the first replicate and 24 suckling mice in the second replicate with 6 negative controls for each replicate**. Un-infected suckling mice of the same age were used as the negative control group The virus dilutions of the neat virus, 10^{-2} and 10^{-4} used in the experimental infection were quantified by plaque assay as follows: - $5.0x10^6$, $2.7x10^4$ and $1x10^3$

remove bold font. change to regular font

remove bold font

PFU/ml respectively. **All animal welfare considerations were taken to minimize suffering or distress. Mice were fed *adlibitum* All mice were observed twice daily until humane endpoint or the end of the 21-day monitoring period. Specific criteria (humane endpoint) included immobility or paralysis (41). Any moribund mice were separated, euthanized by cervical dislocation and preserved at -80^{0}C immediately**. Brain samples were harvested and homogenized in 1ml of cell culture media and a plaque assay test was done to confirm virus infection in the mice.

remove bold font

Plaque reduction neutralization test

The serological assay was done on 324 human sera. The serum samples were heat inactivated at 56°C for 30 minutes and ten -fold dilutions from 1:20 to 1:320 were tested for neutralizing antibodies by a 90% plaque reduction neutralization test ($PRNT_{90}$) in Vero cells. The virus was diluted to a standard concentration that gave at least 50 plaques. The serum dilutions were mixed with the standard concentration of the diluted virus and incubated for 1 h at 37°C. The virus–antibody mixture was inoculated on confluent Vero cells and incubated for 1 h for virus adsorption. After the adsorption, 2.5% methylcellulose 4000 CV (Sigma) mixed with 2X MEM, was added into the wells (32). At day 6 post infection, the plaques were fixed using 3.7% formaldehyde (Sigma) and stained with 0.5% crystal violet (Sigma) in absolute ethanol. Serum samples were considered positive by $PRNT_{90}$ when serum dilution of 1:20 or greater reduced the viral formation of plaques by at least 90% (42).

Data management

All data was entered into an excel database. The sero-prevalence data was imported into Stata version 17.0 for analysis (Stata Corp, College Station, TX). Proportions positive for virus were

compared across socio-demographic characteristics using Chi-square test. All tests were performed at 5% significance level.

Results

Tick abundance and virus characterization

A total of 2342 ticks were collected and pooled to 409 pools representing 9 species after identification. The most abundant species identified was *Hyalomma marginatum* (40%, 162/409), followed by *Amblyomma gemma* (26%, 105/409), *Rhipicephalus pulchellus* (22.4%, 92/409) and *Hyalomma truncatum* (9%, 38/409). Other species identified included *Rhipicephalus appendiculatus*, *Hyalomma dromedarii*, *Amblyomma variegatum*, *Hyalomma albiparmatum* and *Boophilus* spp. Ticks were predominantly collected from camels (73.1%, 299/409) and cattle (26.2%, 107/409). An isolate from a homogenate of *A. gemma* tick species, induced cytopathic effects between 3-7 days post-infection. The isolate was negative by PCR for flaviviruses, orthobunyaviruses and alphaviruses. It was also negative for Dhori, Thogoto, Dugbe and CCHFV tick-borne viruses. Primer sequences utilized for screening viruses in this study are provided (**S1 Table**).

Next generation sequencing

Full genome sequences for the virus indicated that the virus was related to Guertu virus with percent nucleotide identities of approximately 80.42% in the L segment, 76.54% in the M segment and 81.09% in the S segment. The genome architecture was a 3-segmented single stranded RNA and total size of 11847bp with nucleotides lengths of 6403, 3332 and 1752 in the Large (L), Medium (M) and S (Small) segments respectively. The amino acid identity scores for the different genes encoded by the virus were 93.33% for RdRp, 87.36% in the glycoprotein, 83.86% for the nonstructural protein and 92.24% for the nucleocapsid gene. Phylogenetic analysis placed the isolated virus in the same, but unique cluster with Guertu virus, which further

clusters in a similar clade with Dabie bandavirus (SFTSV). This observation was consistent across all the four different genes of the virus **(Fig 2)**.

Fig 2: Phylogenetic analysis of viruses in the genus *Bandavirus*. The phylogenies were inferred based on RdRp sequences (A), Glycoprotein (B), Nonstructural protein (C) and Nucleoprotein sequences (D). The virus isolated in the study is highlighted in red.

Prevalence of virus in ticks, cell growth tropism and pathogenicity in mice

The forward and reverse primers with an expected size of 560bp were designed from the L segment of the virus isolate. The forward- CAAGGCTGAGGATTTGGTATCT and reverse – CTGACTGGGCCCTTTCTATTT sequences for this virus were used to screen more ticks from the region. Nevertheless, the virus was not detected in the additional ticks that were tested. However, in-vitro growth analysis showed that Vero E6, Vero CCL-81 and Vero Biken cells were susceptible to this virus. Peak titres of $1.07x10^6$ PFU/ml were reported in Vero Biken at 72 hours, $7.4x10^5$PFU/ml at 48hrs for Vero CCL-81 and $3.4x10^5$PFU/ml at 48hrs for Vero E6 cells. In contrast, virus titres in C636 cells declined after inoculation from $2x10^4$ PFU/ml to $1.3x10^3$PFU/ml over the 7days **(Fig 3)**. On the other hand, all the suckling mice (15/15) infected with the undiluted virus were euthanized within 6-9 days post-infection. The mice exhibited signs of sickness including being moribund, difficulty in breathing, decreased growth, loose skin and uncoordinated movement. Two of the mice were found cannibalized by the dam on day 7. Of the mice infected with 10^{-2} virus dilution, (10/14) were euthanized between 8-10 days post infection and two were cannibalized by the dam on day 8 and day 13. Two of the mice in the first group survived 21 days post- infection while two in the second replicate survived to day 13 and were euthanized accordingly. However, none of the suckling mice (7/7) infected with the 10^{-4}

virus dilution in the first replicate died and were euthanized on day 21. Between 8-10 days post-infection, the mice appeared to be inactive and sickly with ruffled fur but seemed to recover after day 11. In the second replicate, the suckling mice (6/6) infected with the 10^{-4} virus dilution, were cannibalized by the dam between day 1 -3 post infections **(Fig 4)**. Mouse brain samples that were harvested were quantified by plaques and titres ranged from 1.1 $x10^{6}$ PFU/ml from mice brains inoculated with neat virus to as low as $1x10^{4}$ PFU/ml for mice brains inoculated with10^{-2} diluted virus.

Fig 3. One-step growth curves of Kinna virus in Vero E6, Vero CCL-81 and C636 at MOI of 1, supernants were harvested at the indicated time points.

Fig 4. Survival curves of 1-2 day old Swiss Albino suckling mice inoculated intracerebrally with neat, 10^{-2} and 10^{-4} virus dilutions.

Sero-prevalence in humans

The plaque reduction neutralization test was carried out on 324 human samples. Participants were drawn from Garbatula (n=158) and Merti (n=166) sub counties in Isiolo and their age varied from 14-90 years. Antibodies against the virus were detected in 125 (38.6%, 95% CI 33.3–44.1%) of the samples tested. The $PRNT_{90}$ titres ranged from 125 for the virus at 1:20 dilution, 25 (1:40), 17(1:80), 10(1:160) and 3 (1:320). Notably, all the neutralizing titres at 1:320 were detected in serum samples from women aged 25, 60 and 70 years, the former two being farmers and the latter a housewife. The prevalence of the virus was significantly higher in Garbatula (51.9%) than in Merti (25.9%; $p<0.001$). Most of the sero-positive samples were from people who had resided in the villages for most of their lives. The prevalence was significantly higher in those who had contact with cattle ($p = 0.011$) (**Table 1**). The prevalence generally

increased with age (**Table 2**) with a majority of those positive at dilutions 1:80 or higher being in the 55–90 years age bracket.

Table 1. Virus prevalence by socio-demographic characteristics

Characteristic	Number of participants	Number positive	% positive (95% CI)	Chi square p-value
Overall	324	125	38.6 (33.3–44.1)	
Sub county				<0.001
Garbatula	158	82	51.9 (43.8–59.9)	
Merti	166	43	25.9 (19.4–33.3)	
Village				<0.001
Garfasa	68	40	58.8 (46.2–70.6)	
Kinna	90	42	46.7 (36.1–57.5)	
Bulesa	30	12	40.0 (22.7–59.4)	
Dadachabasa	74	20	27.0 (17.4–38.6)	
Korbesa	62	11	17.7 (9.2–29.5)	
Sex				0.337
Female	210	77	36.7 (30.1–43.6)	
Male	114	48	42.1 (32.9–51.7)	
Age group in years				0.313
14–24	39	14	35.9 (21.2–52.8)	
25–34	68	22	32.4 (21.5–44.8)	
35–44	48	16	33.3 (20.4–48.4)	
45–54	55	20	36.4 (23.8–50.4)	
55–64	42	17	40.5 (25.6–56.7)	
65–90	72	36	50.0 (38.0–62.0)	
Occupation				0.552
Farmer	201	83	41.3 (34.4–48.4)	
Herdsman	95	31	32.6 (23.4–43.0)	
Housewife	16	6	37.5 (15.2–64.6)	

Other	12	5	41.7 (15.2–72.3)	
Contact with chicken				0.070
No	187	80	42.8 (35.6–50.2)	
Yes	137	45	32.8 (25.1–41.4)	
Contact with goats				0.807
No	46	17	37.0 (23.2–52.5)	
Yes	278	108	38.8 (33.1–44.9)	
Contact with cows				0.011
No	105	51	48.6 (38.7–58.5)	
Yes	219	74	33.8 (27.6–40.5)	
Contact with donkeys				0.964
No	251	97	38.6 (32.6–45.0)	
Yes	73	28	38.4 (27.2–50.5)	
Contact with camels				0.851
No	321	123	38.6 (33.3–44.2)	
Yes	3	1	33.3 (1.0–90.6)	
Had previous severe illness with blood symptoms				0.315
No	321	123	38.3 (33–43.9)	
Yes	3	2	66.7 (9.4–99.2)	

Table 2. Number of samples sero-positive for virus at different dilutions and ages

	Age in years						
PRNT result	14–24	25–34	35–44	45–54	55–64	65–90	Total
Negative	25	46	32	35	25	36	199
1:20	14	22	16	20	17	36	125
1:40	0	4	0	3	6	12	25
1:80	0	3	0	2	4	8	17
1:160	0	2	0	0	2	6	10

1:320	0	1	0	0	1	1	3

Discussion

We report the isolation of a virus in the family *Phenuiviridae* and genus *Bandavirus*, tentatively named Kinna virus that is closely related to Guertu virus from a pool of *A. gemma* ticks from Isiolo County. The ICTV has various criteria for the classification of virus species and this differ differs depending on the virus group but includes data on genomic, biological and antigenic properties among other information (43). The species and genus demarcation criteria for *Bandaviruses* requires novel species to have less than 95% sequence identity in the RdRp amino acid sequence (44). Furthermore, based on revisions by Fauquet and Stanley, (2005) (45), tick-borne virus contigs showing greater than 10% difference were considered putative novel species while those with less than 10% difference were identified as strains of known viruses (46). Since the virus isolate had a 93.3% RdRp amino acid sequence identity to Guertu virus while the contigs showed nucleotide similarity (<90%) to Guertu virus, we postulate that this could be a putative novel species.

A high sero-prevalence was reported in the human samples tested against this virus suggesting previous exposure and infection to this virus. Similar to SFTSV, age was a risk factor as those >55years of age reported higher antibodies titres (47). Despite the participants in this study being healthy and few having severe illness with bloody symptoms in the past, the high sero-prevalence reported could be an indication that infection with the virus is mild or asymptomatic. Significant risk factors included sub-county of residence and contact with cattle, with farmers and herdsmen being more predisposed to infection. Herdsmen in particular were also shown to be greatly exposed to Guertu virus than farmers in the Xinjiang province, where the virus was

first isolated (23). Although, the sero-prevalence in our study was not significantly different in males and females male and females, the highest dilution that neutralized this virus was reported in samples collected from women. It is speculated that women spend more time in caring for sick animals than their male counterparts increasing the risk of exposure to diseases (48). We also attributed exposure to high-risk activities such as milking, slaughtering, skinning of dead animals or handling aborting fetuses. Exposure may also have occurred inadvertently from the bite of an infected tick as the domestic animals were infested. Moreover, there could be a possible zoonotic risk associated with this virus due to the fact that the tick vectors reported in this study also prefer wild hosts (49). The probability that this virus may have originated from wild animals is high since the villages in this study area are close to several game reserves that formed zones of human-livestock wildlife convergence.

Mice infected intracerebrally with a high dose of the novel virus did not survive. However, survival rates were high in mice infected with the low dilution of virus suggesting that a lower infectious dose might not be detrimental but may aid in maintaining transmission as these animals act as reservoirs of infection. The dose-dependent effect was demonstrated in a study of mice infected with SFTSV that indicated that severity of infection was a factor of the viral load and that a low amount of virus could result in asymptomatic infection (50). The mice in this study showed signs of clinical disease to Kinna virus and this suggests that the virus could cause disease in humans. The clinical presentation of Guertu virus in humans is not well documented but is believed to be similar to that of *Dabie bandavirus* (23). We hypothesize that the clinical presentation of Kinna virus could be similar to that of Guertu virus as the two viruses share many characteristics including tick vector, ability to infect different cell lines and pathogenicity in mice.

is there any evidence that wild rodents act as reservoirs? If not, then please state that, "rodents could act as reservoirs.."

Previous studies have reported Isiolo county to be a high-risk area for zoonotic diseases such as Rift Valley fever virus, yellow fever virus and *Brucella* spp. (51, 52). These diseases share the same non-specific clinical syndrome with many tick-borne viruses which can lead to misdiagnosis. Thus, the identification of this novel virus from ticks in this county is evidence of the existence of uncharacterized viruses at the human-livestock convergence zones which may be responsible for the burden of undiagnosed febrile illness reported during outbreaks. Our findings, thus underscore the need for increased surveillance to understand the pathology of this new virus, susceptible hosts and reservoirs, and development of appropriate point of care diagnostic tools.

Conclusions

We report the isolation of a novel *Bandavirus* from Isiolo County with a potential to cause disease in human and animal populations. A one-health approach should be considered in the evaluation of the public health significance of this virus and for development of appropriate control strategies. Detection of this new strain also points to the need for continuous monitoring of vector and human populations in high-risk ecosystems in order to provide an update on pathogen diversity.

Acknowledgment

I thank Reuben Lugalia, John Gachoya, Francis Mulwa and Dunstone Beti for their participation in the collection and identification of the ticks used in this study. We are also grateful to Samuel Owaka and Bryson Kimemia for the graphics in this publication.

References

1. Shah T, Li Q, Wang B, Baloch Z, Xia X. Geographical distribution and pathogenesis of ticks and tick-borne viral diseases. Front Microbiol. 2023;14:1185829.
2. Kazimírová M, Thangamani S, Bartíková P, Hermance M, Holíková V, Štibrániová I, et al. Tick-Borne Viruses and Biological Processes at the Tick-Host-Virus Interface. Frontiers in Cellular and Infection Microbiology. 2017;7(339).
3. Bartíková P, Holíková V, Kazimírová M, Štibrániová I. Tick-borne viruses. Acta virologica. 2017;61(4):413-27.
4. Mansfield KL, Jizhou L, Phipps LP, Johnson N. Emerging Tick-Borne Viruses in the Twenty-First Century. Frontiers in Cellular and Infection Microbiology. 2017;7:298-.
5. Zhou H, Xu L, Shi W. The human-infection potential of emerging tick-borne viruses is a global public health concern. Nature Reviews Microbiology. 2023;21(4):215-7.
6. Shi J, Hu Z, Deng F, Shen S. Tick-Borne Viruses. Virologica Sinica. 2018;33(1):21-43.
7. Kuhn JH, Adkins S, Agwanda BR, Al Kubrusli R, Alkhovsky SV, Amarasinghe GK, et al. 2021 Taxonomic update of phylum Negarnaviricota (Riboviria: Orthornavirae), including the large orders Bunyavirales and Mononegavirales. 2021.
8. Zhou CM, Yu XJ. Unraveling the Underlying Interaction Mechanism Between Dabie bandavirus and Innate Immune Response. Frontiers in immunology. 2021;12:676861.
9. Yu X-J, Liang M-F, Zhang S-Y, Liu Y, Li J-D, Sun Y-L, et al. Fever with Thrombocytopenia Associated with a Novel Bunyavirus in China. New England Journal of Medicine. 2011;364(16):1523-32.
10. Saito T, Fukushima K, Umeki K, Nakajima K. Severe fever with thrombocytopenia syndrome in Japan and public health communication. Emerg Infect Dis. 2015;21(3):487-9.
11. Kim KH, Yi J, Kim G, Choi SJ, Jun KI, Kim NH, et al. Severe fever with thrombocytopenia syndrome, South Korea, 2012. Emerg Infect Dis. 2013;19(11):1892-4.
12. Tran XC, Yun Y, Van An L, Kim SH, Thao NTP, Man PKC, et al. Endemic Severe Fever with Thrombocytopenia Syndrome, Vietnam. Emerg Infect Dis. 2019;25(5):1029-31.
13. Liu J-W, Zhao L, Luo L-M, Liu M-M, Sun Y, Su X, et al. Molecular Evolution and Spatial Transmission of Severe Fever with Thrombocytopenia Syndrome Virus Based on Complete Genome Sequences. PLoS One. 2016;11(3):e0151677.
14. Casel MA, Park SJ, Choi YK. Severe fever with thrombocytopenia syndrome virus: emerging novel phlebovirus and their control strategy. Experimental & Molecular Medicine. 2021;53(5):713-22.
15. Fang X, Hu J, Peng Z, Dai Q, Liu W, Liang S, et al. Epidemiological and clinical characteristics of severe fever with thrombocytopenia syndrome bunyavirus human-to-human transmission. PLOS Neglected Tropical Diseases. 2021;15(4):e0009037.
16. Ye C, Qi R. Risk factors for person-to-person transmission of severe fever with thrombocytopenia syndrome. Infection Control & Hospital Epidemiology. 2020;42(5):582-5.
17. Zhuang L, Sun Y, Cui XM, Tang F, Hu JG, Wang LY, et al. Transmission of Severe Fever with Thrombocytopenia Syndrome Virus by Haemaphysalis longicornis Ticks, China. Emerg Infect Dis. 2018;24(5):868-71.
18. Chen C, Li P, Li K-F, Wang H-L, Dai Y-X, Cheng X, et al. Animals as amplification hosts in the spread of severe fever with thrombocytopenia syndrome virus: A systematic review and meta-analysis. International Journal of Infectious Diseases. 2019;79:77-84.
19. Niu G, Li J, Liang M, Jiang X, Jiang M, Yin H, et al. Severe fever with thrombocytopenia syndrome virus among domesticated animals, China. Emerg Infect Dis. 2013;19(5):756-63.
20. Wang S, Li J, Niu G, Wang X, Ding S, Jiang X, et al. SFTS virus in ticks in an endemic area of China. The American journal of tropical medicine and hygiene. 2015;92(4):684-9.

21. Esguerra EM. Heartland Virus: A New Virus Discovered in Missouri. Missouri medicine. 2016;113(4):256-7.

22. Yusufu M, Abula A, Jiang B, Zhumabai J, Deng F, Li Y, et al. Fine mapping of the antigenic epitopes of the Gc protein of Guertu virus. PLoS One. 2022;17(7):e0271878.

23. Shen S, Duan X, Wang B, Zhu L, Zhang Y, Zhang J, et al. A novel tick-borne phlebovirus, closely related to severe fever with thrombocytopenia syndrome virus and Heartland virus, is a potential pathogen. Emerging Microbes & Infections. 2018;7(1):1-14.

24. Matsuno K, Weisend C, Travassos da Rosa APA, Anzick SL, Dahlstrom E, Porcella SF, et al. Characterization of the Bhanja serogroup viruses (Bunyaviridae): a novel species of the genus Phlebovirus and its relationship with other emerging tick-borne phleboviruses. Journal of virology. 2013;87(7):3719-28.

25. Zakham F, Albalawi AE, Alanazi AD, Truong Nguyen P, Alouffi AS, Alaoui A, et al. Viral RNA Metagenomics of Hyalomma Ticks Collected from Dromedary Camels in Makkah Province, Saudi Arabia. Viruses. 2021;13(7):1396.

26. Zohaib A, Zhang J, Saqib M, Athar MA, Hussain MH, Chen J, et al. Serologic Evidence of Severe Fever with Thrombocytopenia Syndrome Virus and Related Viruses in Pakistan. Emerg Infect Dis. 2020;26(7):1513-6.

27. Ergunay K, Mutinda M, Bourke B, Justi SA, Caicedo-Quiroga L, Kamau J, et al. Metagenomic Investigation of Ticks From Kenyan Wildlife Reveals Diverse Microbial Pathogens and New Country Pathogen Records. Frontiers in Microbiology. 2022;13.

28. Ogola EO, Kopp A, Bastos ADS, Slothouwer I, Marklewitz M, Omoga D, et al. Jingmen Tick Virus in Ticks from Kenya. Viruses. 2022;14(5):1041.

29. Mkutu K. Security dynamics in conservancies in Kenya:The case of Isiolo County: BICC Bonn International Centre for Conflict Studies; 2020 [updated 17 Jun 2020; cited 2023 03 Nov 2023]. 13]. Available from: https://www.bicc.de/uploads/tx_bicctools/BICC_Working_Paper_3_2020_01.pdf.

30. Karesh WB, Dobson A, Lloyd-Smith JO, Lubroth J, Dixon MA, Bennett M, et al. Ecology of zoonoses: natural and unnatural histories. The Lancet. 2012;380(9857):1936-45.

31. Mati BM, Muchiri JM, Njenga K, de Vries FP, Merrey DJ. Assessing water availability under pastoral livestock systems in drought-prone Isiolo District, Kenya: IWMI; 2006.

32. Tigoi C, Sang R, Chepkorir E, Orindi B, Arum SO, Mulwa F, et al. High risk for human exposure to Rift Valley fever virus in communities living along livestock movement routes: A cross-sectional survey in Kenya. PLOS Neglected Tropical Diseases. 2020;14(2):e0007979.

33. Okello-Onen J, Hassan, SM, Essuman, S. Taxonomy of African ticks.: Icipe Science Press, Nairobi; 1999.

34. Matthysse J, Colbo, MH. The Ixodid ticks of Uganda together with species pertinent to Uganda because of their present known distribution. College Park, MD: Entomological Society of America; 1987.

35. Lwande OW, Lutomiah J, Obanda V, Gakuya F, Mutisya J, Mulwa F, et al. Isolation of tick and mosquito-borne arboviruses from ticks sampled from livestock and wild animal hosts in Ijara District, Kenya. Vector borne and zoonotic diseases (Larchmont, NY). 2013;13(9):637-42.

36. Ochieng C, Lutomiah J, Makio A, Koka H, Chepkorir E, Yalwala S, et al. Mosquito-borne arbovirus surveillance at selected sites in diverse ecological zones of Kenya; 2007 -- 2012. Virol J. 2013;10(1):140.

37. Sang R, Onyango C, Gachoya J, Mabinda E, Konongoi S, Ofula V, et al. Tickborne arbovirus surveillance in market livestock, Nairobi, Kenya. Emerg Infect Dis. 2006;12(7):1074-80.

38. Clark JJ, Gilray J, Orton RJ, Baird M, Wilkie G, Filipe AdS, et al. Population genomics of louping ill virus provide new insights into the evolution of tick-borne flaviviruses. PLOS Neglected Tropical Diseases. 2020;14(9):e0008133.

39. Kumar S, Stecher G, Tamura K. MEGA7: molecular evolutionary genetics analysis version 7.0 for bigger datasets. Molecular biology and evolution. 2016;33(7):1870-4.

40. Mims CA. Intracerebral injections and the growth of viruses in the mouse brain. British journal of experimental pathology. 1960;41(1):52-9.
41. Lim SM, Koraka P, van Boheemen S, Roose JM, Jaarsma D, van de Vijver DA, et al. Characterization of the mouse neuroinvasiveness of selected European strains of West Nile virus. PLoS One. 2013;8(9):e74575.
42. Gomes FA, Jansen AM, Machado RZ, Jesus Pena HF, Fumagalli MJ, Silva A, et al. Serological evidence of arboviruses and coccidia infecting horses in the Amazonian region of Brazil. PLoS One. 2019;14(12):e0225895.
43. Fauquet CM. TAXONOMY, CLASSIFICATION AND NOMENCLATURE OF VIRUSES. Encyclopedia of Virology. 1999:1730-56.
44. ICTV-Reports. The ICTV report on Virus Classification and Taxon Nomenclature: Family Phenuiviridae, genus Bandavirus [cited 2024 3 Aprol 2024]. Available from: https://ictv.global/report/chapter/phenuiviridae/phenuiviridae/bandavirus.
45. Fauquet CM, Stanley J. Revising the way we conceive and name viruses below the species level: A review of geminivirus taxonomy calls for new standardized isolate descriptors. Archives of virology. 2005;150(10):2151-79.
46. Lin Y, Pascall DJ. Characterisation of putative novel tick viruses and zoonotic risk prediction. 2024;14(1):e10814.
47. Park S-J, Kim Y-I, Park A, Kwon H-I, Kim E-H, Si Y-J, et al. Ferret animal model of severe fever with thrombocytopenia syndrome phlebovirus for human lethal infection and pathogenesis. Nature Microbiology. 2019;4(3):438-46.
48. Affognon H, Mburu P, Hassan OA, Kingori S, Ahlm C, Sang R, et al. Ethnic groups' knowledge, attitude and practices and Rift Valley fever exposure in Isiolo County of Kenya. PLOS Neglected Tropical Diseases. 2017;11(3):e0005405.
49. Walker AR, Bouattour A, Camicas J-L, Estrada-Peña A, Horak IG, Latif AA, et al. Ticks of Domestic Animals in Africa: a Guide to Identification of Species: Bioscience Reports, Edinburgh Scotland,U.K.; 2003.
50. Jin C, Liang M, Ning J, Gu W, Jiang H, Wu W, et al. Pathogenesis of emerging severe fever with thrombocytopenia syndrome virus in C57/BL6 mouse model. Proceedings of the National Academy of Sciences of the United States of America. 2012;109(25):10053-8.
51. WHO. Disease Outbreak News; Yellow Fever - Kenya 25 March 2022. Available from: https://www.who.int/emergencies/disease-outbreak-news/item/yellow-fever-kenya.
52. Nyamota R, Maina J, Akoko J, Nthiwa D, Mwatondo A, Muturi M, et al. Seroprevalence of Brucella spp. and Rift Valley fever virus among slaughterhouse workers in Isiolo County, northern Kenya. PLoS Negl Trop Dis. 2023;17(10):e0011677.

Supporting information

S1 Table: A list of conventional PCR primers used in this study

Figure

Click here to access/download;Figure;Fig 1.tif

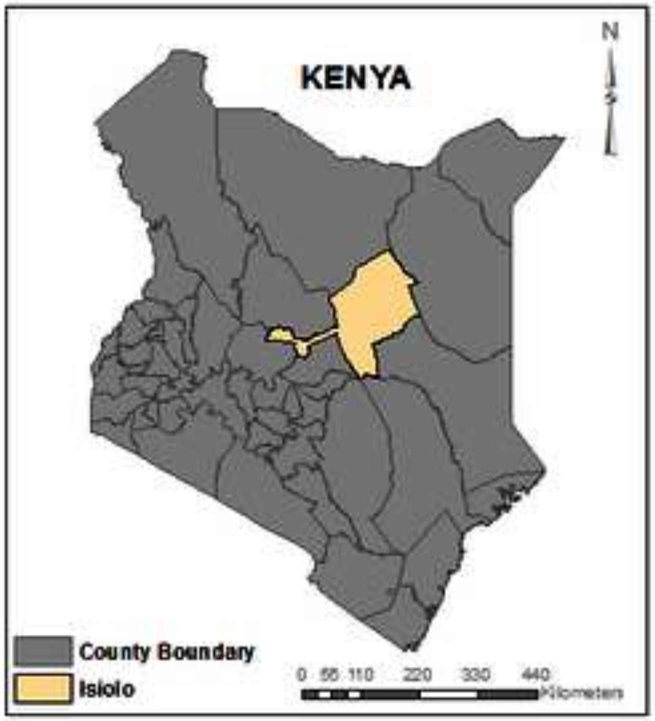

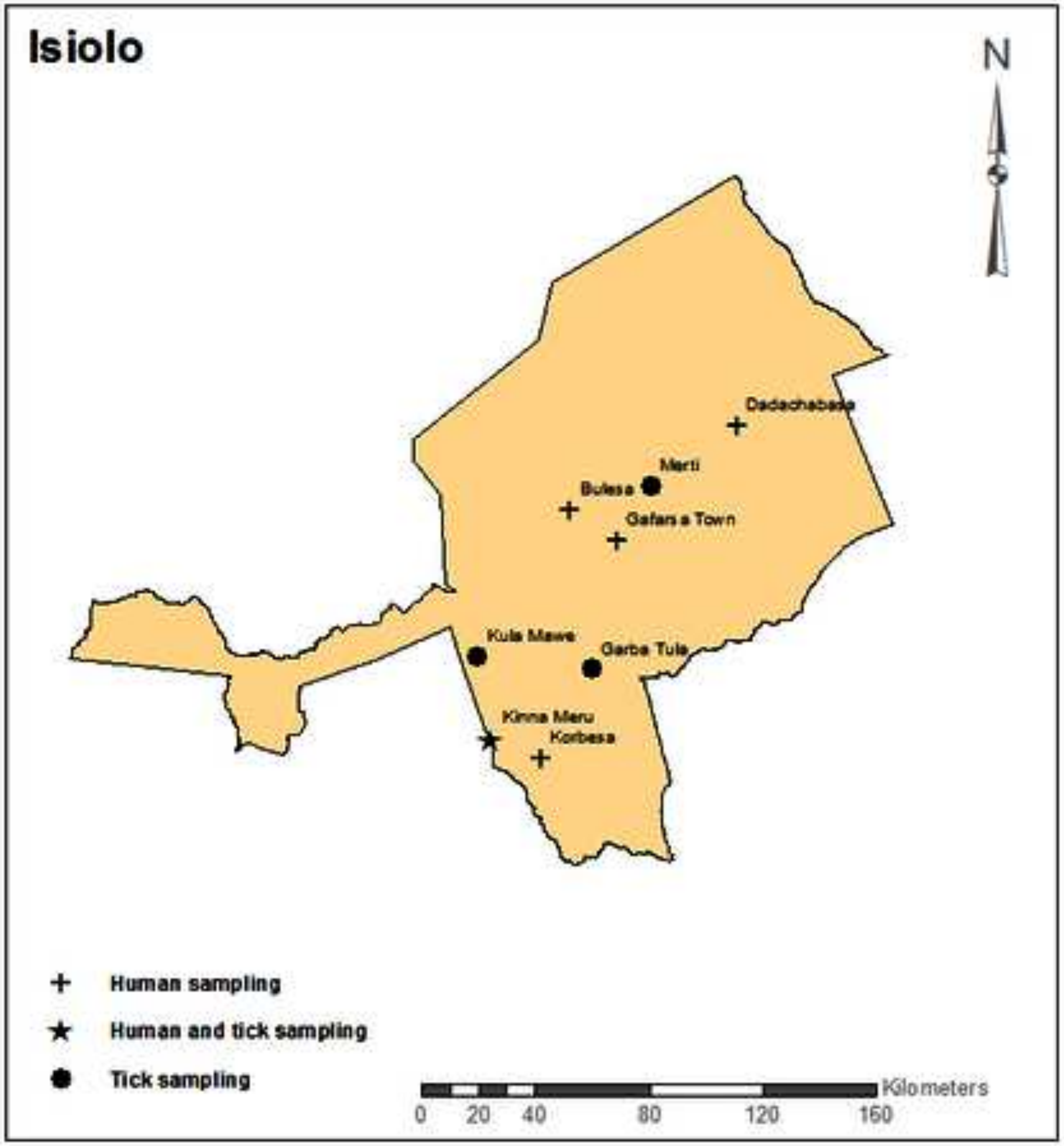

Figure Click here to access/download;Figure;Fig 2.tif

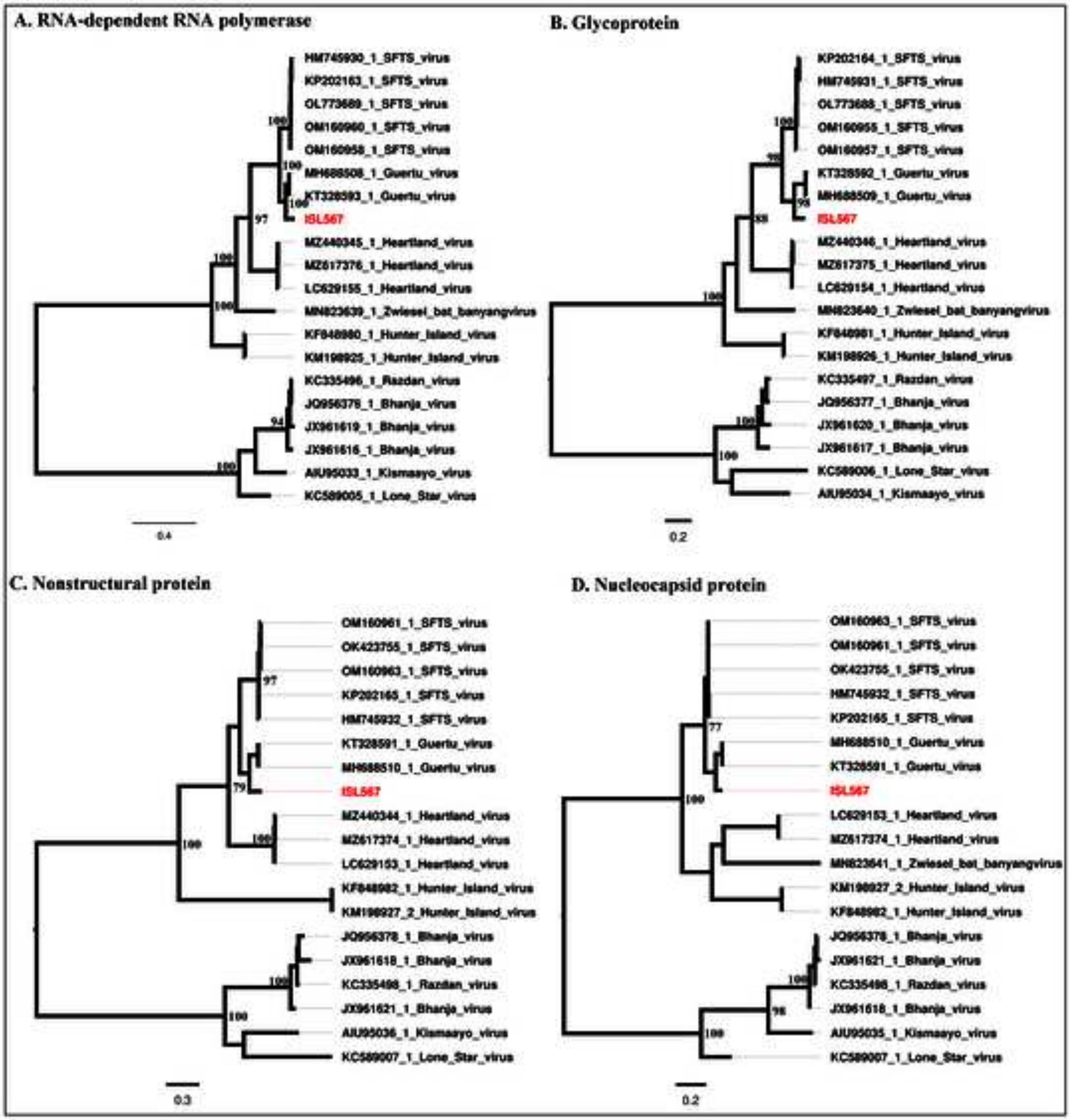

 Click here to access/download;Figure;Fig 3.tif

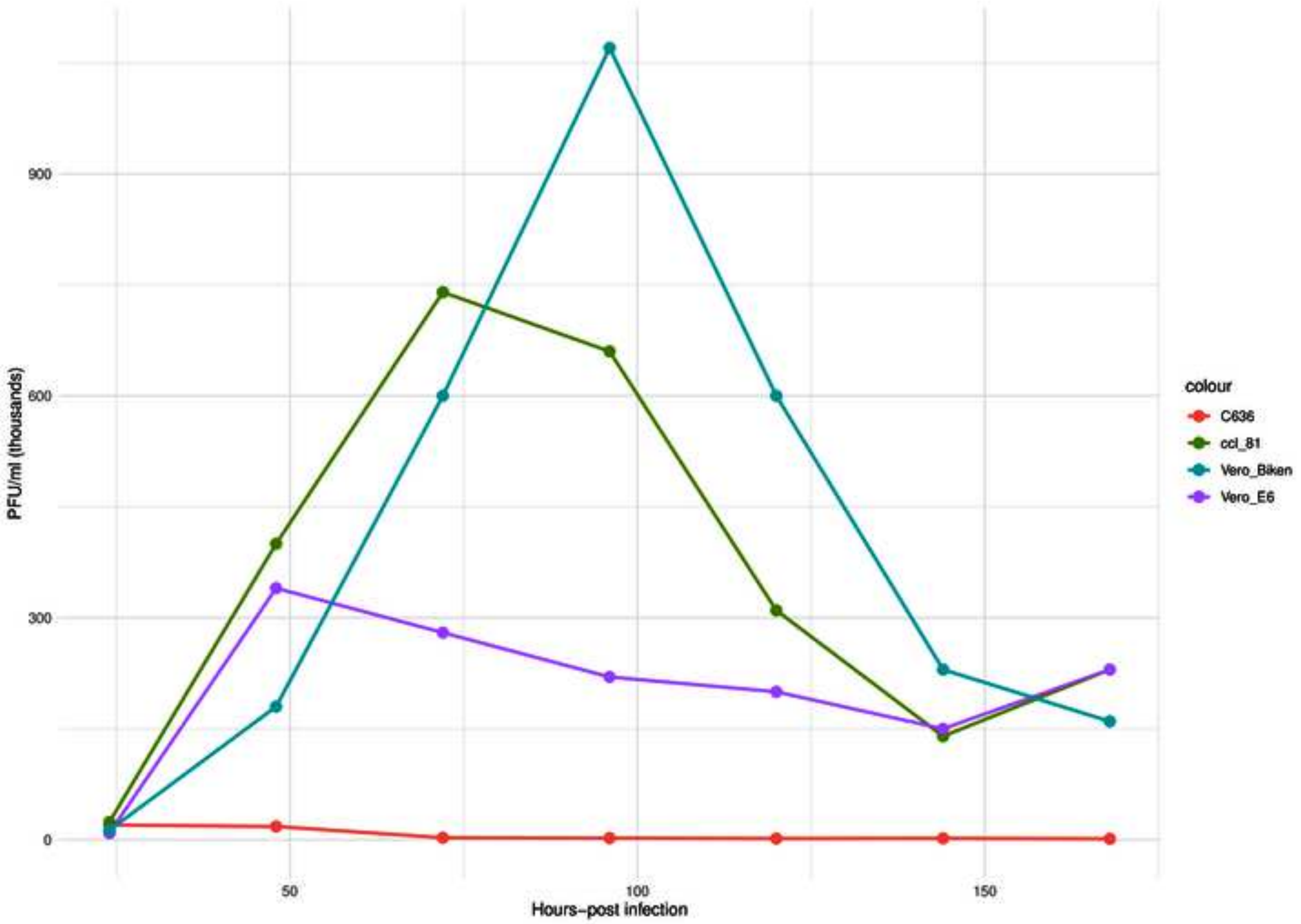

Click here to access/download;Figure;Fig 4.tif

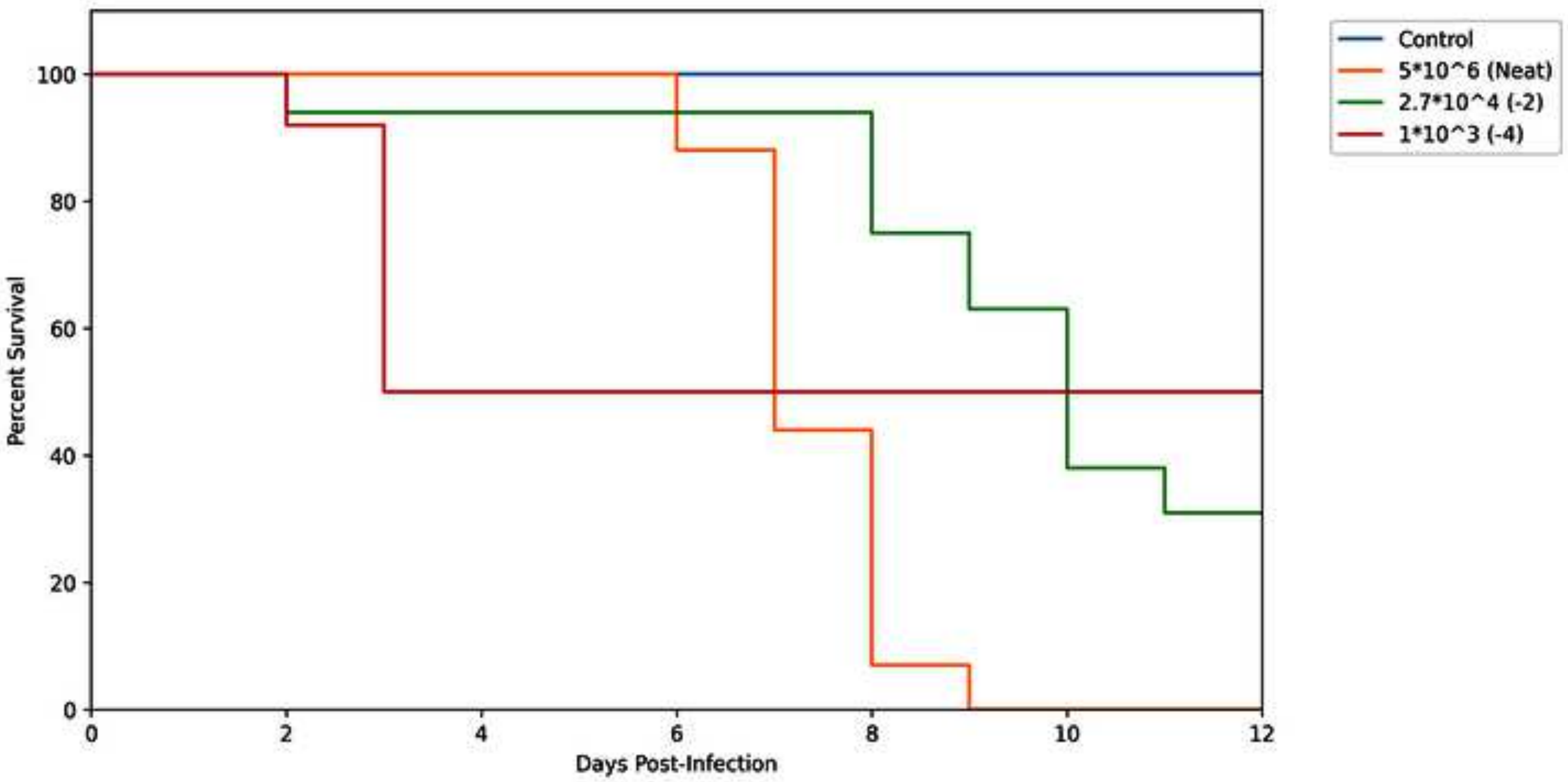

Click here to access/download
Supporting Information
S1_Table.docx
