# Supplementary material for: Detection and prevalence of a novel Bandavirus related to Guertu virus in Amblyomma gemma ticks and human populations in Isiolo County, Kenya
Source: PLoS One. 2024 Sep 20;19(9):e0310862. doi: 10.1371/journal.pone.0310862 (PMC11414941; doi:10.1371/journal.pone.0310862)
Supplement: Attachment — Submitted filename: Responses to reviewers comments Plos one July 2024 .docx [file pone.0310862.s003.docx]

**RESPONSES TO REVIEWER #1 COMMENTS**

1. Review of ‘Detection and prevalence of a novel Bandavirus related to Guertu virus in *Amblyomma gemma* ticks and human populations in Isiolo County, Kenya’. The study looked at the detection of a novel virus belonging to the Bandavirus genus in *Amblyomma* ticks and humans in Kenya. The study is interesting. The study however does not include its limitations. I noted following limitations in the study. Although a large number of ticks were tested, only in one pool the novel virus was detected, whereas the in human population more than 38% has serological evidence of the virus.

**Response: That we found one pool testing positive for the virus at the time, for us was exciting. The number of detections of a virus often depends on its level of circulation at the point of sampling, which is influenced by factors that are not known to us at this point in time, Line 363-366. Detection of one positive pool for us is a very important finding and evidence of circulation of this virus in our region. Serologic evidence of circulation represents possible cumulative exposure of the population over time, an important additional finding. This finding opens opportunity for further in-depth surveillance when funding is available. We also tested an additional pool of 200 tick from this region by PCR but did not detect the novel virus again, Line 184 and Line 267.**

2. I found that the cut-off titre for sero-positives was quite low. According to me, I would have only considered titres from 1:160. Little is also discussed about cross-reactivity with other viruses and false positives. I do not know what is the sensitivity and specificity of the plaque reduction virus neutralization test. This information should have been included in the methods and limitations should have been discussed at the end of the manuscript.

**Response: The cut-off titre for sero-positivity of this novel virus was retained at 1:20 and since the samples were not tested against other viruses in the *Bandavirus* genus, no cross-reactivity was reported. There are no known circulating *Bandaviruses* locally that we could consider for cross-neutralization, Line367-368. The sensitivity and specificity are above 70% and is the reason plaque reduction test is considered a gold standard.**

3. In addition, the study would have had more power if the samples from ticks came from animals that were owned/herded by the humans that were samples. As far as I understood the samples were from same counties but tick and human samples were not linked to same location.

**Response: This suggested sampling plan would have been ideal. The limitation is addressed, Line 366-369. It was after the virus was detected, isolated and characterized that the serologic analysis was considered. For this reason, the human samples were not linked directly to the exact location/herd where the tick samples had been collected. However, some of the human samples were collected from the general locality where the ticks were collected, Fig 1, Line 118 and Line 124. Further, the target communities are largely nomadic and often move through wide areas in search of pasture and water creating potential for movement of animals and ticks over a wider space. This pastoral system thus increases the potential for pathogen transmission in the region. This is also supported by the fact that; the human samples had been collected along the animal migratory routes used by the pastoralists in this region.**

4. In addition, it would have provided more insight if also animals were sampled. This should have been included as limitations of the study.

**Response: Due to the scope of the research and level of funding for the work, no animals were sampled, and this has been captured as a limitation of the study. This initial funding opens opportunity for seeking more funding for such additional studies as suggested by the reviewers, Line 374-377.**

5. Suggestion are made that the novel virus can cause the same symptoms of humans as the related Guertu virus but yet it seems although evidence of past infection was found in a high number of humans but yet those people were healthy at the time of sample.

**Response: The symptoms caused by this virus are not known. There is need for further studies to determine symptoms associated with acute infection of this virus. At this time, we can only speculate that this novel virus may cause similar course of infection as Guertu or *Dabie bandavirus* (SFTSV), Line 371-374. The suggestion has been made to promote awareness among clinicians in our region to enhance the index of suspicion as they review patients, Line 352-353.**

6. Although the sample size is small, I find it odd that the conclusion was made that the virus could cause serious disease in humans yet there was no evidence because people were healthy although had been infected previously based on serological evidence.

**Response: Please refer to the response in point 5. Although, the symptoms are not known, the virus was also shown to infect mice and thus we speculate that the virus may have potential to cause disease in humans, Line 346-348.**

7. The route of exposure is also not well understood yet. Are humans exposed through adult *Amblyomma* ticks from accidental close contact with cows or are do they acquire infection from the immature stages directly from environment. For example, African tick bite fever is transmitted more likely from the immature stages of *Amblyomm*a ticks.
I think above issues and limitations could be added and discussed in the paper.

**Response: The route of exposure is not well understood, it could be through the bite of the tick or through handling of infectious materials from infected animal,** **Line 334-338.**

8. For the remainder, I think this is a very interesting paper.

**Response: We appreciate the review comments and the concerns raised which we have used to improve the manuscript. Thank you.**

**RESPONSE TO REVIEWER #2 COMMENTS**

1. This study isolates and characterizes a type of novel Bandavirus. Experiments to determine its pathogenicity as well as prevalence in humans were performed. The virus could have potentially important health consequences in humans. Authors perform rigorous experimental methods and conclude that this virus is closely related to Guertu virus within the genus. This is an important paper that needs to be published.

I have added minor comments in a marked-up text.

**Response: We thank reviewer number 2 for the specific comments on the document that were very valuable in improving the manuscript. We have done our best to respond to all of them. Thank you.**

**RESPONSES TO EDITORS COMMENTS**

1. Please ensure that your manuscript meets PLOS ONE's style requirements, including those for file naming. The PLOS ONE style templates can be found at https://journals.plos.org/plosone/s/file?id=wjVg/PLOSOne_formatting_sample_main_body.pdf and https://journals.plos.org/plosone/s/file?id=ba62/PLOSOne_formatting_sample_title_authors_affiliations.pdf

**Response: These guidelines have been met**

2. To comply with PLOS ONE submissions requirements, in your Methods section, please provide additional information regarding the experiments involving animals and ensure you have included details on (1) methods of sacrifice, (2) methods of anesthesia and/or analgesia, and (3) efforts to alleviate suffering.

**Response: Additional information has been provided for experiments involving animals. Line 214-217**

3. When completing the data availability statement of the submission form, you indicated that you will make your data available on acceptance. We strongly recommend all authors decide on a data sharing plan before acceptance, as the process can be lengthy and hold up publication timelines. Please note that, though access restrictions are acceptable now, your entire data will need to be made freely accessible if your manuscript is accepted for publication. This policy applies to all data except where public deposition would breach compliance with the protocol approved by your research ethics board. If you are unable to adhere to our open data policy, please kindly revise your statement to explain your reasoning and we will seek the editor's input on an exemption. Please be assured that, once you have provided your new statement, the assessment of your exemption will not hold up the peer review process

**Response: Data sharing plan revised**

4. Please include your full ethics statement in the ‘Methods’ section of your manuscript file. In your statement, please include the full name of the IRB or ethics committee who approved or waived your study, as well as whether or not you obtained informed written or verbal consent. If consent was waived for your study, please include this information in your statement as well.

**Response: Ethics statement was added, Line 131-133.**

5. We note that Figure 1 in your submission contain map/satellite images which may be copyrighted. All PLOS content is published under the Creative Commons Attribution License (CC BY 4.0), which means that the manuscript, images, and Supporting Information files will be freely available online, and any third party is permitted to access, download, copy, distribute, and use these materials in any way, even commercially, with proper attribution. For these reasons, we cannot publish previously copyrighted maps or satellite images created using proprietary data, such as Google software (Google Maps, Street View, and Earth). For more information, see our copyright guidelines: <http://journals.plos.org/plosone/s/licenses-and-copyright>.

**Response: The map was created from the ARC GIS software by Samuel Owaka and details are provided under the figure caption for Fig 1.**

6. Please review your reference list to ensure that it is complete and correct. If you have cited papers that have been retracted, please include the rationale for doing so in the manuscript text, or remove these references and replace them with relevant current references. Any changes to the reference list should be mentioned in the rebuttal letter that accompanies your revised manuscript. If you need to cite a retracted article, indicate the article’s retracted status in the References list and also include a citation and full reference for the retraction

**Response: The references were formatted and checked for completeness. One reference was added, Line 482.**
