# Supplementary material for: Detection and prevalence of a novel Bandavirus related to Guertu virus in Amblyomma gemma ticks and human populations in Isiolo County, Kenya
Source: PLoS One. 2024 Sep 20;19(9):e0310862. doi: 10.1371/journal.pone.0310862 (PMC11414941; doi:10.1371/journal.pone.0310862)
Supplement: Attachment — Submitted filename: Responses to reviewers comments Plos one 30 Aug 2024 .docx [file pone.0310862.s004.docx]

**RESPONSES TO EDITORS COMMENTS**

1. We note that Figure 1 in your submission contain map/satellite images which may be copyrighted. All PLOS content is published under the Creative Commons Attribution License (CC BY 4.0), which means that the manuscript, images, and Supporting Information files will be freely available online, and any third party is permitted to access, download, copy, distribute, and use these materials in any way, even commercially, with proper attribution. For these reasons, we cannot publish previously copyrighted maps or satellite images created using proprietary data, such as Google software (Google Maps, Street View, and Earth). For more information, see our copyright guidelines: <http://journals.plos.org/plosone/s/licenses-and-copyright>.

We require you to either (a) present written permission from the copyright holder to publish these figures specifically under the CC BY 4.0 license, or (b) remove the figures from your submission:

**Response: Thank you for this comment. The maps were not copyrighted. I have edited the caption to Figure 1 as follows:**

**Fig 1.** Map of Isiolo County showing the sites where tick and human samples were collected in this study. Base maps, boundaries and shape files of Kenyan map and administrative boundaries of the County and Sub-county were derived from GADM data version 4.1 (<https://gadm.org>) and the maps were generated using ArcGIS Version 10.2.2 (<http://desktop.arcgis.com/en/arcmap>) advanced license) courtesy of Samuel Owaka.
