# Supplementary material for: Detection and prevalence of a novel Bandavirus related to Guertu virus in Amblyomma gemma ticks and human populations in Isiolo County, Kenya
Source: PLoS One. 2024 Sep 20;19(9):e0310862. doi: 10.1371/journal.pone.0310862 (PMC11414941; doi:10.1371/journal.pone.0310862)
Supplement: Attachment — Submitted filename: Responses to reviewers comments Plos one 5 Sep 2024 (1).docx [file pone.0310862.s005.docx]

**RESPONSES TO REVIEWER #1 COMMENTS**

1. Thanks for addressing the comments I had diligently and adding a limitations section. I have just one small comment re: line 305-307, I recommend to use sero-prevalence/sero-positivity instead of prevalence because the humans were tested by serology.

Thanks for this interesting study. I accepted the revised manuscript for publication.

**Response: The revision of the word prevalence to sero-prevalence / sero-positivity was done, Line 305-307. We appreciate your review comments as they greatly improved this manuscript. Thank you.**

**RESPONSES TO EDITORS COMMENTS**

1. Please review your reference list to ensure that it is complete and correct. If you have cited papers that have been retracted, please include the rationale for doing so in the manuscript text, or remove these references and replace them with relevant current references. Any changes to the reference list should be mentioned in the rebuttal letter that accompanies your revised manuscript.

**Response: The references were checked for completeness.**
